# What are the triggers for palliative care referral in burn intensive care units? Results from a qualitative study based on healthcare professionals’ views, clinical experiences and practices

**DOI:** 10.1177/02692163241229962

**Published:** 2024-02-19

**Authors:** André Filipe Ribeiro, Sandra Martins Pereira, Rui Nunes, Pablo Hernández-Marrero

**Affiliations:** 1 Faculdade de Medicina da Universidade do Porto, Porto, Portugal; 2 Centro Hospitalar de Entre o Douro e Vouga, Santa Maria da Feira, Portugal; 3 Universidade Católica Portuguesa, CEGE: Research Center in Management and Economics – Ethics and Sustainability Research Area, Católica Porto Business School, Porto, Portugal; 4 International Network UNESCO Chair in Bioethics, Porto, Portugal; 5 Portuguese Nurses Association for Long-Term and Palliative Care (AECCP), Lisbon, Portugal

**Keywords:** Palliative care, burns, critical care, referral, triggers, qualitative research

## Abstract

**Background:**

Burns are a global public health problem, accounting for around 300,000 deaths annually. Burns have significant consequences for patients, families, healthcare teams and systems. Evidence suggests that the integration of palliative care in burn intensive care units improves patients’ comfort, decision-making processes and family care. Research is needed on how to optimise palliative care referrals.

**Aim:**

To identify triggers for palliative care referral in critically burned patients based on professionals’ views, experiences and practices.

**Design:**

Qualitative study using in-depth interviews.

**Setting/participants:**

All five Burn Intensive Care Units reference centres across Portugal were invited; three participated. Inclusion criteria: Professionals with experience/working in these settings. A total of 15 professionals (12 nurses and 3 physicians) participated. Reflexive thematic analysis was performed.

**Results:**

Three main triggers for palliative care referral were identified: (i) Burn severity and extension, (ii) Co-morbidities and (iii) Multiorgan failure. Other triggers were also generated: (i) Rehabilitative palliative care related to patients’ suffering and changes in body image, (ii) Family suffering and/or dysfunctional and complex family processes, (iii) Long stay in the burn intensive care unit and (iv) Uncontrolled pain.

**Conclusions:**

This study identifies triggers for palliative care in burn intensive care units based on professionals’ views, clinical experiences and practices. The systematisation and use of triggers could help streamline referral pathways and strengthen the integration of palliative care in burn intensive care units. Research is needed on the use of these triggers in clinical practice to enhance decision-making processes, early and high-quality integrated palliative care and proportionate patient and family centred care.

**What is already known about the topic?**

-  End-of-life care is paramount in the care provided to critically burned patients who are dying and to their families.
-  Palliative care provision requires a specific set of competencies to enable improved quality-of-life, comfort and optimal family support.
-  End-of-life care and the integration of palliative care in burn intensive care units are characterised by complexity and uncertainty but rarely studied.

**What this paper adds?**

-  Main triggers for palliative care in burn intensive care units were identified: Burn severity and extension, co-morbidities and multiorgan failure.
-  Additional triggers were also generated: Rehabilitative palliative care related to patients’ suffering and changes in body image, family suffering and/or dysfunctional and complex family processes, long stay in burn intensive care units and uncontrolled pain.

**Implications for practice, theory or policy**

-  The systematisation and use of triggers for palliative care referral in burn intensive care units could help streamline referral pathways and support the integration of palliative care in burn intensive care units with quality and effectiveness.
-  The identification of these triggers could enable the provision of the most adequate care, contributing to the optimisation of available health resources.
-  Further research is needed on the use of these triggers in clinical practice to enhance decision-making processes, timely referral, high-quality integrated palliative care and proportionate patient and family-centred care.

## Background

Burns are complex injuries that can result in lifelong physical and psychological scarring. They can be an extremely traumatic experience, which is associated with pain and potential (unbearable) suffering both for patients and their families. Mental health, quality-of-life, ability to return to work and mortality, all are influenced by the experience of severe burns.^1,2^ Burn-related wounds can represent a chronic condition, placing a high burden not only on patients’ families but also on national healthcare systems globally, requiring long-term care and medical support from different specialties.^3–6^

Severe burns often threaten patients’ lives.^7^ End-of-life care, which refers specifically to the comprehensive care for dying patients in the last few hours, days or weeks of life,^8^ is thus a major step in the care provided to critically ill burned patients. In fact, patients with no prospect of cure should experience more comfortable and peaceful end-of-life care.^9^ According to the literature, burn specialists have long recognised the need for and have role modelled a comprehensive approach incorporating relief of distress as part of care during critical illness.^10^ Nevertheless, while evidence suggests that the integration of palliative care in burn intensive care units improves patients’ comfort, decision-making processes and family care,^11^ it also shows that palliative care is rarely involved in the care provided to these patients.^12^

The International Society for Burn Injuries^13^ considers palliative care a continuation of treatment once futile medical treatment is observed. This is challenging since futility is often perceived as a ‘grey zone’ where goals of care might be difficult to define^14^ and treatments that improve patients’ comfort and minimise suffering of both patients and their families are as important as those aimed at saving patients’ lives.^15–17^ For severe conditions, the American Burn Association suggests the use of a triage-to-benefit ratio,^18,19^ where age and total burn surface percentage are considered to estimate patients’ survival probability and introduce palliative care for those who are unlikely to survive. Although relevant, this only accounts for a small number of critically burned patients who could benefit from palliative care. Four criteria groups for palliative care consultations were distinguished: clinical risk factors, changes in clinical trajectory, decision-making process and communication needs.^20^ However, despite meeting these criteria, most patients did not receive palliative care at admission, and findings were limited by its retrospective nature.

Studies highlight the need for timely and high-quality palliative and end-of-life care in the trajectories of critically ill burned patients. For instance, early deaths have been reported to occur within 72 h of admission to the burn unit^17,21–23^ with late deaths occurring after a period of active treatment (usually in the order of days or weeks).^17,23^ Therefore, the decision to provide comfort care or end-of-life care should be made with the family as soon as practically possible,^17,24^ and the decision to make a palliative care referral might be challenging.

Palliative care is still suboptimal in burn intensive care units.^12,17^ A recent review suggests that further research is needed to (i) better understand how the specific set of palliative competencies can enable improved quality-of-life, comfort and optimum family support and (ii) study the best way to provide optimal end-of-life care and foster integrated palliative care in burn intensive care units.^12^ Identifying triggers for palliative care referral in burn intensive care units could help to better integrate palliative care when needed.

## Aim

To identify triggers for palliative care referral in critically burned patients based on professionals’ views, experiences and practices.

## Methods

This study embraced a constructivist paradigm since it intended to understand the phenomenon under study, triggers for palliative care referral in burn intensive care units. Meanings were built and interpreted based on participants’ views, experiences and practices.^25–30^ The knowledge generated was the result of a constructive process, focussing on the analysis of processes or functions of person-situation-interaction, in which we sought to obtain comprehensive detail to be confident that we were representing differing and complementary views.^27,31^

A qualitative exploratory study was conducted using in-depth interviews. This design is particularly relevant where information is required directly from those experiencing the phenomenon under investigation.^28,29,32–34^ It is a valuable approach in healthcare research to gain deeper insight into professionals’ experiences, practices and sense-making around them.^27,30,33–35^ The Standards for Reporting Qualitative Research (SRQR) was used as reporting guideline.^36^

### Context

This study is part of a wider project entitled InPalIn-B©: Integrating Palliative Care in Burn Intensive Care Units that aims at improving palliative care for critically burned patients in Portugal. All five Burn Intensive Care Units reference centres across Portugal were invited to participate.

### Population

Professionals working in the five Burn Intensive Care Units reference centres across Portugal. The sole inclusion criterion was to have experience or to work in one of these burn intensive care units as this would constitute an information-rich ‘key informant’, someone who has specialised knowledge that would otherwise be unavailable or difficult to access.^37–40^ No exclusion criteria were considered as long as the inclusion criteria would be met.

### Sampling

Purposive sampling techniques were used to maximise understanding of the phenomena under investigation and ensure variety and inclusion of information-rich^40,41^ participants (professional groups, years of professional experience, years of experience in burn intensive care, gender and education in palliative care, critical care and burn care) from different Burn Intensive Care Units reference centres across Portugal.

### Recruitment

One researcher (A.F.R.) contacted all the five Burn Intensive Care Units reference centres in Portugal. After this initial contact, A.F.R. presented the project to the director of the three participating burn intensive care units. Following this, professionals’ recruitment was made through a publicised invitation in each burn intensive care unit, explaining the purpose and objectives of the study, the relevance of their participation and the potential benefits resulting from the study. This recruitment strategy was considered as the most suitable for the participant units.

Telephone and email contacts were established with potential participants to provide more detailed information about the project. Data collection was scheduled according to each participant’s availability and preference.

### Data collection

In-depth interviews were performed as this is the most appropriate method for providing rich data surrounding professionals’ knowledge and experience. A topic guide was developed in conformity with the interview guide from a previous project on the integration of palliative care in intensive care (InPalIn©). The research team of Project InPalIn-B© has a wealth of combined palliative care, health services research and clinical experience in intensive care and burn units. The topic guide was iteratively modified as necessary to ensure follow-up with topics in subsequent interviews (Supplemental Box 1).

Interviews were conducted by one researcher (A.F.R.) together with one or two of the other researchers (either S.M.P. or P.H.M. or both). This was deemed to be appropriate to encompass a variety of experiences relevant to conduct these interviews (e.g. clinical experience in burn intensive care units and plastic surgery; managerial and organisational experience; and palliative care). Power balance was ensured by explaining the reasons and asking each participant beforehand about the possibility of having more than one interviewer. As all the interviewers had relevant clinical experience, no objections were made by any of the participants. None of the researchers had prior relationships with any of the participants.

Due to the COVID-19 pandemic, interviews were conducted via Zoom® and lasted between 30 and 120 min (mean of 54 min). Interviews were completed from July to October 2020. Participant recruitment ceased when no new themes became apparent.^30,42,43^

To ensure reflexivity, three researchers (A.F.R., S.M.P. and P.H.M.) documented reflections after each interview. These were discussed at regular team meetings throughout the data collection period and contributed to a deep, engaged and critically open reflexivity throughout the research process.^30^

### Data analysis

Audio-recordings were transcribed verbatim by A.F.R. Transcripts were validated by S.M.P. and P.H.M., as appropriate. A thematic analysis was performed to the transcripts of interviews to identify, analyse and report patterns (themes) within the data.^30,42,44,45^ QSR^®^ NVivo 12 was used to store, organise and support the analysis of pseudonymised data.^46^ Drawing on Braun and Clarke’s six-phase guide in reflexive thematic analysis,^42,45^ an inductive approach was employed, coupled with the researchers’ existing knowledge, to progressively refine themes based on codes identified in the data. Acknowledging the research team’s experiences and beliefs influencing the interpretive analysis of the data, we remained mindful of this and invited all participant perspectives to be captured.^27,42,45^ The operationalisation of the six phases of reflexive thematic analysis is presented in Table 1. Th-roughout these six phases, ongoing discussions were held within the research team that embraced a critical dialogue until reaching interpretative depth and reflexive practice. This process facilitated the capture of different perspectives to enhance the analytical framework. Table 2 systematises the measures undertaken during the research process to ensure methodological rigour.^42,44,45,47–49^

**Table 1.** Operationalisation of Braun and Clarke’s^42^ six phases of reflexive thematic analysis.

Six phases of reflexive thematic analysis^42^	Operationalisation of the six phases of reflexive thematic analysis
Phase 1. Familiarising yourself with the dataset	Initially, the three authors (A.F.R., S.M.P. and P.H.M.) read and reread the transcripts to gain a sense of participants’ perspectives and experiences.
Phase 2. Coding	Following the scrutiny of the transcripts conducted during Phase 1, one researcher (A.F.R.) coded the data, detailing inductive descriptive codes by marking similar phrases or words from the participants and developed a codebook. The codebook was shared and discussed with the other two researchers (S.M.P. and P.H.M.). This allowed for more reflexivity and consistent interpretation and classification of the data.
Phase 3. Generating initial themes	The shared codebook was used to guide coding of remaining transcripts by A.F.R., who collated the codes and identified where some of them merged into themes. All codes and themes were independently analysed by two researchers (A.F.R. and S.M.P.).
Phase 4. Developing and reviewing themes	Consecutive rounds of discussions were held among all researchers (A.F.R., S.M.P. and P.H.M.), enhancing reflexivity and generating a thematic ‘map’ of the analysis.
Phase 5. Refining, defining and naming themes	An ongoing and collaborative analysis was performed by all researchers (A.F.R., S.M.P. and P.H.M.) to refine each theme and sub-themes and build the overall ‘story’ that could answer the research question.
Phase 6. Writing up	A concise, coherent and logical account of the ‘story the data tell’, within and across themes, was produced by all researchers (A.F.R., S.M.P. and P.H.M.), allowing for a final opportunity to analyse and relate back to the research question and literature.

**Table 2.** Measures undertaken during the research process to ensure methodological rigour^28,29,42,44,45,47–49^.

Dimensions of methodological rigour in qualitative research	Measures undertaken in this research
Confirmability: • Reflexivity • Triangulation	1. Interviewers created reflexive journals and took personal notes during all interviews. The latter were shared and discussed in briefing meetings among three members of the research team (A.F.R., S.M.P., P.H.M.) seeking enhanced reflexivity and interpretative depth. 2. Researchers shared a codebook, which allowed for reflexivity and consistent interpretation and classification of the data. 3. Researchers’ existing knowledge and experience was acknowledged as part of the research process. 4. The following triangulation techniques were applied: • *Data triangulation*, using data from different clinical settings (three burn intensive care units based on three different hospitals and cities) and participant professional (15 professionals: 12 nurses and 3 physicians). • *Investigator triangulation*, with multiple researchers involved together in collecting and analysing data (A.F.R., S.M.P. and P.H.M.). These researchers have different previous experiences as healthcare professionals (e.g. intensive care, emergency, burn intensive care units and palliative care).
Transferability: • Purposive sampling	*Purposive sampling* *Maximum variation (or heterogeneous) sampling*: 1. All the reference burn intensive care units in Portugal were invited to collaborate in our research. 2. Within the three participant units, all professionals were invited to participate in an interview. 3. A second round of invitations for interviews was performed in each unit. 4. Different professional groups. 5. Variability in the years of professional experience and experience working in burn intensive care. *Homogeneous sampling* 1. Only reference centres of burn intensive care units recognised by the Portuguese Ministry of Health were invited to participate. *Expert sampling* 2. Participants are considered experts in their field (i.e. burn intensive care). Participants were considered key informants (i.e. someone who has specialised knowledge that would otherwise be unavailable or difficult to access).

### Ethics procedures

Project InPalIn-B© received ethics clearance from the Research Ethics Lab of the Institute of Bioethics [Ref.09/2018]. Ethics approval was obtained from all participant institutions. Each participant provided informed consent, which included the recording of the interview. Data protection procedures were observed; assurances of confidentiality were provided via pseudonymisation.

## Results

### Characteristics of participants

**Figure 1.**
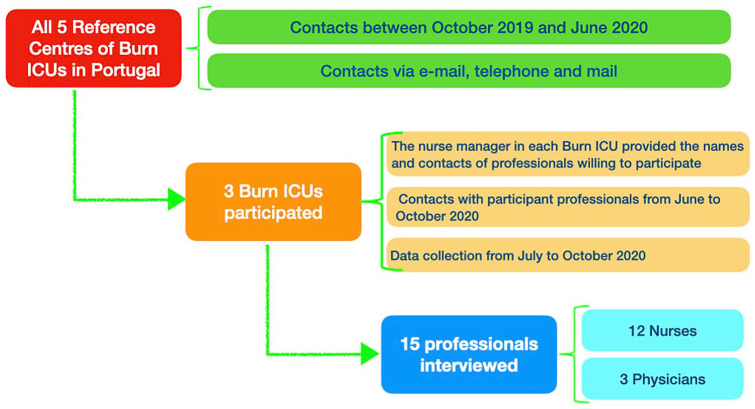
Illustrates the study flowchart.

Out of five Burn Intensive Care Units, three agreed to participate. Only physicians (*n* = 3) and nurses (*n* = 12) with different specialties (the three physicians were plastic surgeons; four nurses were specialists in medical-surgical nursing and one in rehabilitation) accepted the invitation to participate in this study. Eleven were female and four males, with ages ranging from 29 to 59 years; median age of 39 years. The median professional experience with critically burned patients was 6 years, ranging from 1 to 31 years. Two participants had a managerial role. Only one participant had post-graduate education in palliative care (Figure 1).

Two main themes were generated: *Main triggers for palliative care referral in Burn Intensive Care Units* (Burn ICUs) that were identified by all the participants and *Additional triggers for palliative care referral in Burn ICUs* that were identified by some participants.

### Main triggers for palliative care referral in burn ICUs

The three main triggers for palliative care referral id-entified by all the participants were: (i) Burn severity and extension, (ii) Co-morbidities and (iii) Multiorgan failure.

Participants considered that burn severity, namely the presence of third degree burns and/or having more than 80% of burned body surface, was a trigger for palliative care referral.

*‘The severity of burns could be considered a factor to ask for the support from the palliative care team. . . for instance, patients with third degree burns. . . and also depending on their location. . .’* (Interviewee 7)

*‘It would basically be this: (. . .) the percentage of body surface burned (. . .) someone with 80% or more of burned body surface would certainly benefit from palliative care.’* (Interviewee 15)

Co-morbidities such as cancer, organ failure, metabolic failure and dementia were also considered by all participants as a main trigger, particularly when the patient was of older age (i.e. aged 80 years and above).

*‘I think that, for instance, if the critically burned patient has cancer, we could refer to palliative care (. . .)’* (Interviewee 2)

*‘(. . .) the presence of comorbidities.’* (Interviewee 15)

*‘Perhaps when the patient gets into metabolic failure. . .’* (Interviewee 7)

*‘For example, if the burned patient has dementia. . . this could be a situation in which it could be helpful to get their* [palliative care] *support.’* (Interviewee 3)

*‘(. . .) age. . . very old patients. . . aged 80 and above who usually have other comorbidities. . .’* (Interviewee 15).

Multiorgan failure due to severe burns was also considered a main trigger for palliative care referral. In participants’ perspective, this would mean that the patient was in need for end-of-life care, facing a life-threatening condition.

*‘(. . .) multiple organ failure; failure of the organs, which could mean that the patient is facing the end-of-life and therefore could benefit from palliative care. In case of multiple organ failure, which threatens the patient’s life, this situation almost always arises, almost always. . .’* (Interviewee 1)

### Additional triggers for palliative care referral in burn ICUs

Four additional triggers for palliative care referral in Burn ICUs were also identified: (i) Rehabilitative palliative care related to patients’ suffering and changes in body image, (ii) Family suffering and/or dysfunctional and complex family processes, (iii) Long stay in the Burn ICU and (iv) Uncontrolled pain.

For some participants, critically burned patients had major changes in their body image that cause unbearable suffering. This situation requires not only long-term rehabilitative care but also palliative care, which can alleviate suffering.

*‘Thinking already of a prospect for discharge, because a burned patient will require a lot of care, namely rehabilitative care (. . .) skin care treatments, such as special clothing, skin protection; and therefore, if someone, the palliative care team could give this support (. . .).’* (Interviewee 1)

*‘These patients suffer so much. . . their body image can be emotionally and also socially devastating. . . perhaps in these cases, palliative care could help. . .’* (Interviewee 3)

Family suffering and/or dysfunctional and complex family processes were also mentioned by some participants as potential triggers for palliative care referral in Burn ICUs. Participants were aware that families are part of the care process and suffer immediately when they know about a patient’s accident and its consequences. Severe critically burned patients who are cared for in Burn ICUs face isolation. Often, families are prevented from having physical contact with burned patients due to the risk of sepsis. As the focus of professionals working in Burn ICUs is the patient, sometimes the family might not be seen as in need of care. This could be addressed through the involvement of palliative care teams. Participants recognised that these teams could provide emotional and/or social support throughout hospitalisation in Burn ICUs.

*‘The family always ends-up being forgotten. (. . .) At the beginning, the risk of death is enormous and sometimes families cannot even identify their loved one. This is brutal. They* [palliative care] *could provide help in these situations.’* (Interviewee 4)

Some professionals considered long stays in Burn ICUs as a trigger for palliative care. In line with their expe-rience, perspective and practice, critically burned patients sometimes remain hospitalised for very long periods of time. During this time, the critical condition (severe burns that threaten patients’ lives) becomes chronic and patients require long-term care that could be provided by a palliative care team.

*‘The burned person is a chronic patient who needs care forever. The intensity of care may diminish, but these patients always have to be cared for. Some patients stay with us for nine, ten months. . . sometimes a year. . . these patients will require long-term care that perhaps could be provided by a palliative care team. . .’* (Interviewee 7).

Finally, uncontrolled pain was another additional trigger for palliative care referral. This was mainly stated by physicians who considered that palliative care teams have a relevant knowledge on how to manage pain and other symptoms.

*‘We are usually quite good managing symptoms, such as pain. But if I think about situations in which I would refer patients to palliative care, then it would be for uncontrolled pain. . .’* (Interviewee 6).

Besides the two themes and correspondent sub-themes that refer to the three main and four additional triggers for palliative care referral in Burn ICUs, other themes which can be linked to triggers also emerged from the analysis. These refer to: (i) impossibility to identity triggers for palliative care referral in Burn ICUs, (ii) respect for individuality and person-centred care, (iii) uncertainty, (iv) quality-of-life and (v) preparing for discharge.

### Impossibility to identity triggers for palliative care referral in burn ICUs

Two participants could not identify or think about potential triggers for palliative care referral in Burn ICUs. These professionals lacked awareness about the role of palliative care within their organisation. One of these two professionals did not even know that a palliative care team existed in his/her organisation.

*‘I cannot say. I did not even know that we have a palliative care team in our hospital.’* (Interviewee 14)

### Respect for individuality and person-centred care

Some professionals reflected on the palliative care referrals as personalised decisions, focussed on individual patients and their needs.

*‘It seems to me that, as with everything in our work and in our profession, we would have to take into account the person standing in front of us. And so, I think the primacy would be to contextualize that person.’* (Interviewee 5)

### Uncertainty

According to the participants, the evaluation of patients’ needs occurs as soon as possible after their admission into the Burn ICU. However, clinical situations in Burn ICUs tend to change rapidly and are characterised by complexity and uncertainty. This requires an ongoing and constant evaluation and flexibility in the care process. Sometimes, uncertainty might also hamper the referral to palliative care.

*‘. . .when we have patients getting worse, maybe we won’t have time to act and benefit from these teams* [palliative care].*’* (Interviewee 14)

### Quality-of-life

Some participants reflected on the risk for low quality-of-life that critically burned patients might experience after being discharged from Burn ICUs. In their perspective, improving the quality-of-life of these patients could be addressed through the involvement of palliative care.

*‘And that patient, for example, that survives, but will practically not have any kind of relationship or relational life. . . This could also be one of those situations where palliative care could be involved.’* (Interviewee 10)

### Preparing for discharge

**Figure 2.**
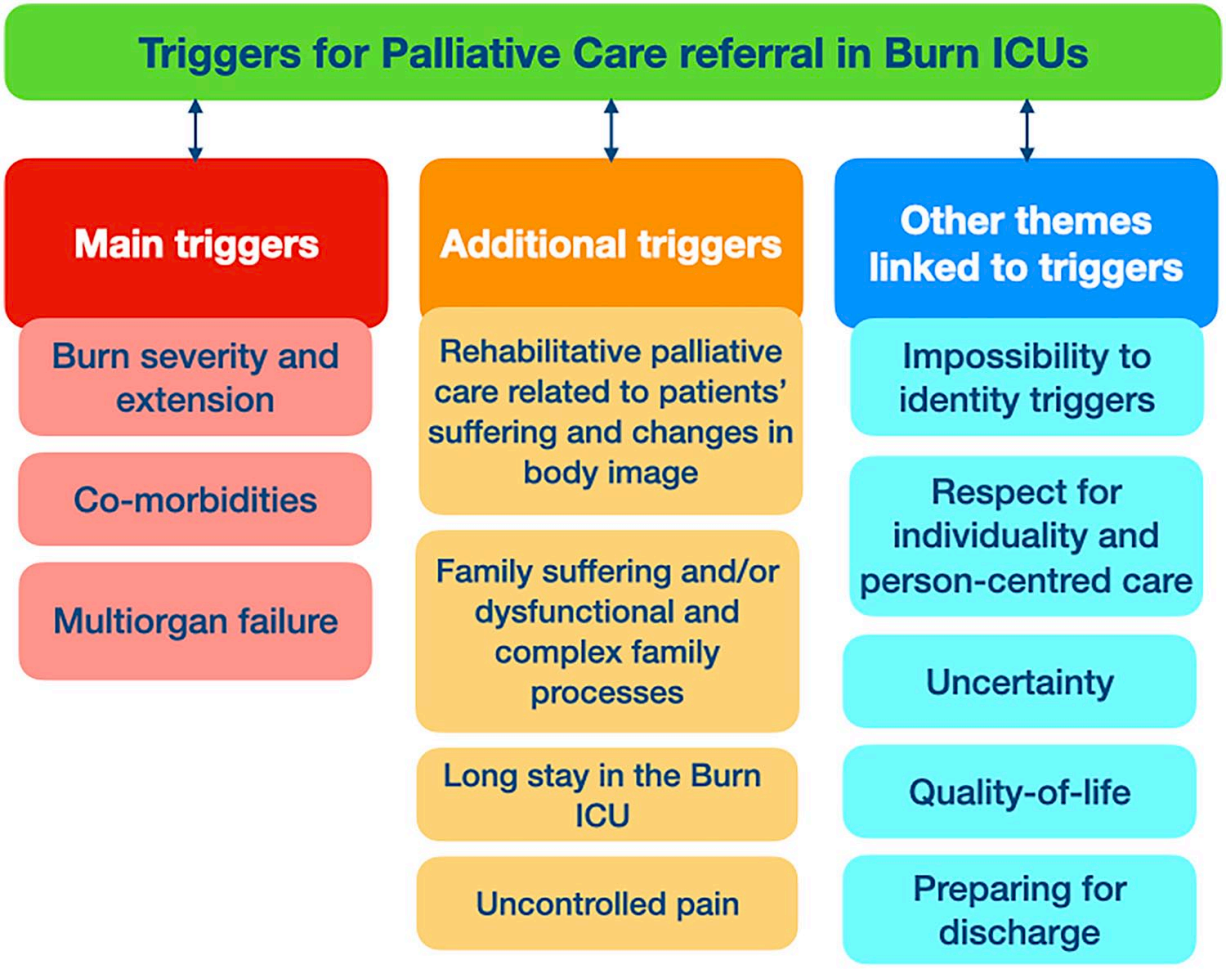
Systematises the main findings of this study.

Although preparing for discharge was not considered a trigger for palliative care referral in Burn ICUs, participants well-thought-out that this situation could foster a potential collaboration between burn intensive care and palliative care teams. Participants reflected on the need to identify the best place of care after discharge and how palliative care could be involved in this decision-making process (Figure 2).

*‘Thinking already of a prospect for discharge, because a burned patient will require a lot of care that often is not even cheap. (. . .) Nowadays, there is an absence of any support and coordination. I think these situations would also be worthy to be signalled, perhaps to palliative care or other support teams.’* (Interviewee 5)

## Discussion

### Main findings of the study

Our findings suggest that there are three main triggers for palliative care referral of critically burned patients in Burn Intensive Care Units (Burn ICUs): Burn severity and extension, Co-morbidities and Multiorgan failure. Four additional triggers were also identified, namely: Need for rehabilitative palliative care, Family suffering, Long stay in Burn ICUs and Uncontrolled pain. These triggers were identified based on the views, experiences and practices of professionals working in Burn ICUs, focussing on factors that could be important to consider when identifying the need for palliative care in critically burned patients.

Findings are aligned with the international literature about referral criteria to palliative care, particularly in ICUs^16,50–55^ and show the complexity of this integration. In fact, professionals consider palliative care within an extensive frame of action, applicable throughout the trajectory of critically burned patients. This can occur from admission to discharge and can also include rehabilitative palliative care after discharge. To promote a successful integration, organisational aspects need to be taken into account. These may vary across healthcare systems and organisations, making this integration particularly complex.

Besides the identification of triggers, our study also suggests the need to consider other features, which can be linked to triggers, to ensure high-quality palliative care for critically and severely burned patients. Respecting patients’ individuality and providing person-centred care, focussing on the quality-of-life and preparing for discharge were also highlighted.

Clinical complexity and uncertainty made it also difficult (or even impossible) for some professionals to identify triggers for palliative care referral. Previous studies have shown that the end-of-life period in Burn ICUs is poorly defined coupled with prognostic uncertainty.^9,56^ Collaborative models could better address clinical uncertainty and complexity, supporting the integration of palliative care in burn intensive units and helping professionals working in this context to seek out for palliative care consultations when their care becomes futile or in conditions around end-of-life decisions and care.^24,56–58^

### What this study adds?

While the main triggers for palliative care referral in burn intensive care units are described in textbooks,^13,18,19^ the additional triggers that were generated are particularly interesting and novel. By moving the focus from comfort care at the end-of-life after forgoing life-sustaining treatments to rehabilitative palliative care related to patient suffering and changes in body image, family suffering and/or dysfunctional and complex family processes, our study expands existing knowledge.

Participants’ perspectives are in line with the conceptual evolution of palliative care, acknowledging that palliative care represents significantly more than end-of-life care.^8,59^ This can make a difference for patients, families and professionals, promoting a comprehensive and individualised support from the moment of admission in a burn ICU until discharge, which can often occur after very long periods of hospitalisation. The physical (e.g. uncontrolled pain), psychosocial (e.g. unbearable suffering and changes in body image) and spiritual support can also be determinant after discharge. This is aligned with previous studies that emphasised how severely burned patients on the recovery pathway may have biopsychosocial needs, such as body image/altered self-concept and pain during rehabilitation, requiring palliative care.^17,60^

**Figure 3.**
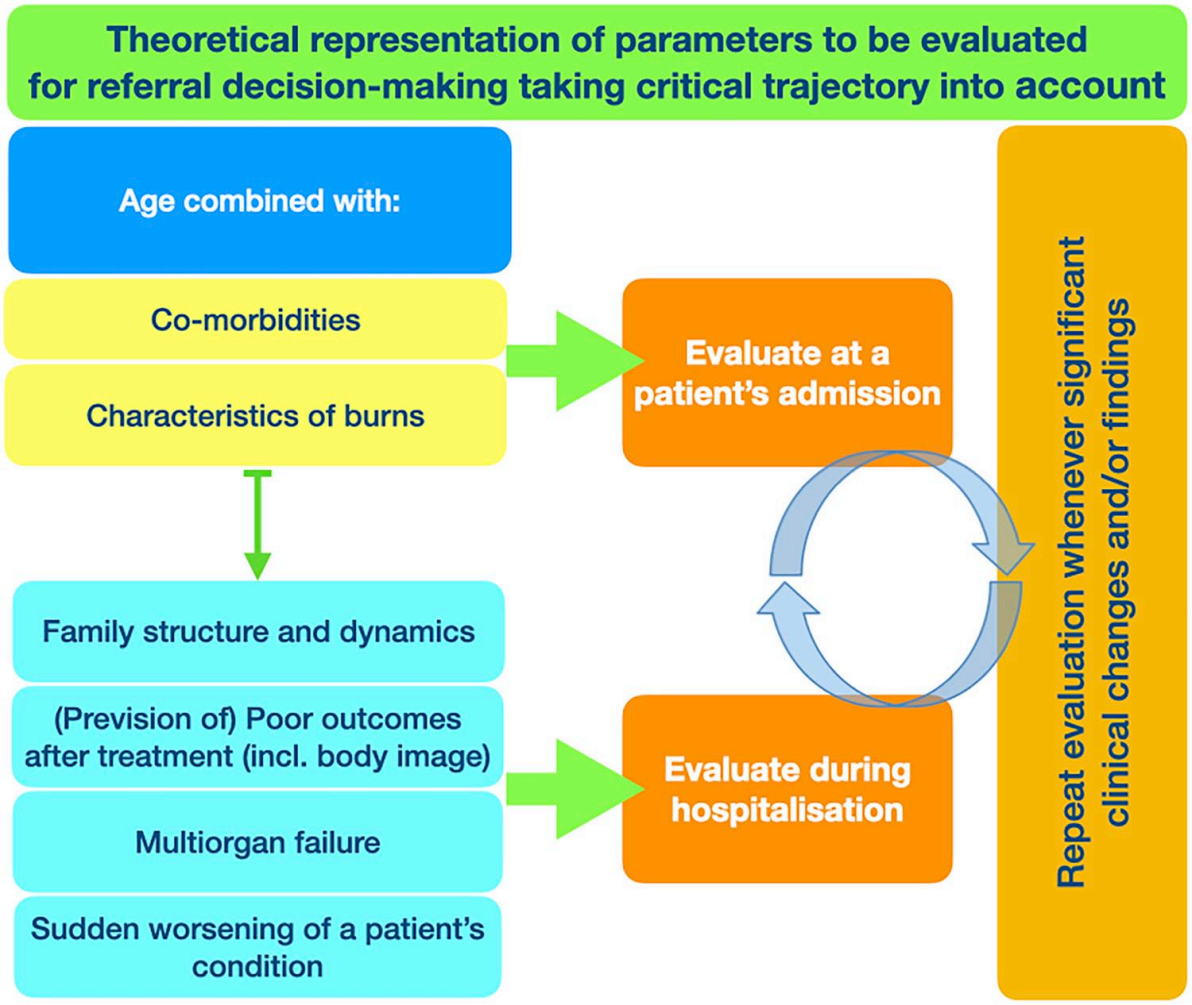
Theoretical representation of parameters to be evaluated for referral decision-making taking critical trajectory into account.

Taking this clinical complexity and uncertainty into account, as well as our findings and their interpretation, the triggers for palliative care referral in Burn ICUs need to be considered in light of patients’ trajectories, which include: (i) Patients’ previous condition; (ii) the characteristics of burn injury(ies); and (iii) the prevision of outcomes (Figure 3). This requires a continuous and ongoing clinical evaluation, during which professionals need to repeat a patient’s evaluation whenever significant clinical changes or findings are encountered.

In what refers to patients’ previous condition, major burned patients are more likely to survive in Burn ICUs when they are younger and do not have comorbidities.^7^ Indeed, the presence of comorbidities is a strong clinical indicator of possible complications during intensive care treatment. It is also significantly related to increased lengths of stay and a higher likelihood of death.^61,62^ Patients with HIV/AIDS, metastatic cancer, renal and/or liver disease are at very high risk for mortality when compared with patients without those diseases who have similar burn injuries.^61^ Over the last decades, medical and surgical intensive care management strategies of severely burned patients have had major scientific developments. Nowadays, patients who are aged below 55 and suffer severe burn and inhalation injuries can survive.^63^ Considering comorbidities as a trigger, anamnesis and clinical assessment assume a determinant role at patients’ admission. They ensure an accurate assessment of patients’ clinical history and previous health problems, their actual needs and healing potential, leading to cost-effective diagnostics and treatments.^64^

It is also of foremost importance to properly determine the characteristics of burn injuries, as wound treatment is associated to the overall outcome of treatments and care provided to these patients. The prognosis also depends on the severity of the initial burn injury and on any further complications.^65^ An accurate assessment of the burn severity is determinant for both wound care and the establishment of the right treatments.^66^ Burns are complex and represent great risk for patients, namely the risk of infection and sepsis and the risk of multiple organ failure, resulting in high mortality and even higher morbidities.^65,66^ Wounds associated to burns demand a specific care programme that respects their slow progress and changing nature over a timeframe that can go from months to even years in the case of massive injuries.^67^ After evaluating the type of burn, professionals need to evaluate their extension, usually expressed as the percentage of total body-surface area burned and describe burn depth. Location is another important factor to consider in association with changes in body image.^68,69^

The prevision of outcomes is another element that needs to be considered, trying to determine the characteristics of patients’ lives after discharge. Some validated scales can be helpful. For example, the Injury Severity Score is built on the basis of worst injury of six body systems and is often used in trauma situations. When applied to burns, this score should combine age and total body surface area burned to better predict patients’ probabilities of mortality or morbidity.^70^ Measuring the quality-of-life is also important to qualify the subjective burden of burns in patients surviving burn accidents.

The variety of triggers that emerged in this study warrants a more in-depth discussion. On the one hand, it shows the lack of recognition of condition-specific triggers which can help to signal that a palliative care approach would be appropriate.^71^ On the other hand, it constitutes a major challenge and can be associated to the clinical complexity and uncertainty that characterises severe and critically burns.

In line with our findings, needs-based trigger systems seem to be the optimal approach as they enhance the identification of palliative care needs by any professional and facilitate the referral whenever needed. As palliative care is, per definition, based on patients’ needs,^72^ this needs-based trigger system has also been suggested for other contexts (e.g. oncology and neurology).^73–76^

### Strengths and limitations

To the best of our knowledge, this is the first empirical study that identifies triggers for palliative care referral in burn intensive care units based on professionals’ clinical experience, views and practices. The fact that the identification of these triggers is made by those same professionals who can refer these patients to palliative care might facilitate the implementation of this trigger system in clinical practice.

Yet, our study is not without limitations. First, this is the first qualitative exploratory narrative study on this highly relevant topic. Second, it only includes the perspective of a limited number of professionals, mostly nurses. While nurses are at the forefront of healthcare provision and a core element in Burn ICUs teams, highly involved in end-of-life decision-making,^77–80^ some caution is needed in the interpretation of these findings. Therefore, the use of these triggers in clinical practice requires further research, consensus and standardisation.

## Conclusions

Burn injuries represent a complex, life-threatening and life changing situation in patients’ lives, causing unbearable suffering for patients and their loved ones and a challenge for healthcare professionals, teams and systems. Important systematic and continuous evaluations are needed from the admission in Burn ICUs, so treatment strategies are constantly redefined in a context of major clinical complexity and uncertainty.

This study identifies triggers for palliative care referral in Burn ICUs based on professionals’ views, experiences and practices. The systematisation and use of triggers tools could help streamline referral pathways and strengthen the integration of palliative care in Burn ICUs with high-quality and effectiveness. This could enable the provision of the most adequate care, contributing to the optimisation of available health resources. Further research is needed on the use of these triggers in clinical practice to enhance decision-making processes, timely referral, high-quality integrated palliative care and proportionate patient and family-centred care.

## Supplemental Material

sj-pdf-1-pmj-10.1177_02692163241229962 – Supplemental material for What are the triggers for palliative care referral in burn intensive care units? Results from a qualitative study based on healthcare professionals’ views, clinical experiences and practices Supplemental material, sj-pdf-1-pmj-10.1177_02692163241229962 for What are the triggers for palliative care referral in burn intensive care units? Results from a qualitative study based on healthcare professionals’ views, clinical experiences and practices by André Filipe Filipe Ribeiro, Sandra Martins Pereira, Rui Nunes and Pablo Hernández-Marrero in Palliative Medicine
