# Supplementary material for: What are the triggers for palliative care referral in burn intensive care units? Results from a qualitative study based on healthcare professionals’ views, clinical experiences and practices
Source: Palliat Med. 2024 Feb 19;38(3):297–309. doi: 10.1177/02692163241229962 (PMC10955784; doi:10.1177/02692163241229962)
Supplement: sj-pdf-1-pmj-10.1177_02692163241229962 – Supplemental material for What are the triggers for palliative care referral in burn intensive care units? Results from a qualitative study based on healthcare professionals’ views, clinical experiences and practices [file sj-pdf-1-pmj-10.1177_02692163241229962.pdf]

Box 1. Interview Guide*: Integrating Palliative Care in Burn Intensive Care Units (InPalIn-B©), adapted from Project InPalIn©

Topics and Questions
What is palliative care for you?
What does the integration of palliative care in burn intensive care units mean to you?
When you think about integrating palliative care in other contexts and services, what ideas come up to your mind?
Who do you think could benefit directly or indirectly from the integration of palliative care in a burn intensive care unit? Why and how?
Are there any criteria that could signal a patient admitted to the burn intensive care unit to benefit from palliative care? What would trigger your decision to refer a patient to palliative care? In what situations would you ask for the advice of the palliative care team? When in the critical situation would you make this referral? Why?
Is there anything else that you would like to add about the topic of the integration of palliative care in burn intensive care units?

* The original interview guide is broader and part of a larger study entitled InPalIn-B© about the integration of palliative care in burn intensive care units. The questions presented in this box are the ones used to explore participants' views, experiences, and practices on the topic "Triggers for palliative care referral".
